# Sediment Microbiota as a Proxy of Environmental Health: Discovering Inter- and Intrakingdom Dynamics along the Eastern Mediterranean Continental Shelf

**DOI:** 10.1128/spectrum.02242-22

**Published:** 2023-01-16

**Authors:** Maya Lalzar, Tal Zvi-Kedem, Yael Kroin, Stephane Martinez, Dan Tchernov, Dalit Meron

**Affiliations:** a Bioinformatics Services Unit, University of Haifa, Haifa, Israel; b Morris Kahn Marine Research Station, Faculty of Marine Biology, Leon H. Charney School of Marine Sciences, University of Haifa, Haifa, Israel; University of Minnesota Twin Cities

**Keywords:** eastern Mediterranean, environmental health, microbiome, sediment

## Abstract

Sedimentary marine habitats are the largest ecosystem on our planet in terms of area. Marine sediment microbiota govern most of the benthic biological processes and therefore are responsible for much of the global biogeochemical activity. Sediment microbiota respond, even rapidly, to natural change in environmental conditions as well as disturbances of anthropogenic sources. The latter greatly impact the continental shelf. Characterization and monitoring of the sediment microbiota may serve as an important tool for assessing environmental health and indicate changes in the marine ecosystem. This study examined the suitability of marine sediment microbiota as a bioindicator for environmental health in the eastern Mediterranean Sea. Integration of information from *Bacteria*, *Archaea*, and *Eukaryota* enabled robust assessment of environmental factors controlling sediment microbiota composition: seafloor-depth (here representing sediment grain size and total organic carbon), core depth, and season (11%, 4.2%, and 2.5% of the variance, respectively). Furthermore, inter- and intrakingdom cooccurrence patterns indicate that ecological filtration as well as stochastic processes may control sediment microbiota assembly. The results show that the sediment microbiota was robust over 3 years of sampling, in terms of both representation of region (outside the model sites) and robustness of microbial markers. Furthermore, anthropogenic disturbance was reflected by significant transformations in sediment microbiota. We therefore propose sediment microbiota analysis as a sensitive approach to detect disturbances, which is applicable for long-term monitoring of marine environmental health.

**IMPORTANCE** Analysis of data, curated over 3 years of sediment sampling, improves our understanding of microbiota assembly in marine sediment. Furthermore, we demonstrate the importance of cross-kingdom integration of information in the study of microbial community ecology. Finally, the urgent need to propose an applicable approach for environmental health monitoring is addressed here by establishment of sediment microbiota as a robust and sensitive model.

## INTRODUCTION

Sedimentary marine habitats, which cover most of the ocean’s floor, are the largest ecosystem on our planet in terms of area. This environment is a central component of the marine ecosystem, being responsible for much of the global biogeochemical activity, including carbon, macro- and micronutrient, and trace element cycling (1, 2). The sediment also serves as a source (e.g., via mineralization of organic matter) and a sink (e.g., via carbon sequestration) of organic and mineral materials, thus also regulating the water column’s biogeochemistry and biological activity (3). These important ecosystem services are largely facilitated by the activity of microbiota which inhabit and engineer the sediment environment (4, 5). In benthic processes, microbiota support the base of aquatic food chains, including organic matter remineralization and degradation of pollutants (6).

Many studies have focused on marine microbiota in the water column (7, 8), in symbiosis with marine organisms (9, 10), and in deep-sea sediments (11, 12). In contrast, our knowledge of the sediment microbiota (SM) along the continental shelf (the focal point of human activity), in the eastern Mediterranean Sea specifically, is very limited. Furthermore, the majority of studies focus on *Bacteria*, *Archaea*, or *Eukaryota* separately (13, 14), without considering interkingdom interactions. Such interactions may be important for understanding drivers of microbiota assembly, cohabitation (due to environmental filtering), and even codependency. Additionally, long-term or spatially extensive marine SM studies are rare. Considering the importance of SM for overall ecosystem function, our lack of knowledge requires urgent attention.

Due to their short generation time, high functional diversity, and phenotypic plasticity, microbial communities are highly dynamic and are alert responders to environmental changes. The composition and diversity of SM are directly influenced by a variety of environmental factors, including sedimentation rate, climate, water salinity, organic matter composition, concentration, and flow, sediment type, and pH. For example, substantial differences in composition were observed between SM of the continental shelf, continental slope, and deep basin at the eastern Mediterranean Sea (EMS) (15). In addition, different studies show that SM change in composition in response to many stressors of anthropogenic origin (16, 17). Examples include heavy metals (e.g., cadmium [18] and mercury [19]), chemical pollution (20), polynuclear aromatic hydrocarbons (PAHs) (21), and nutrient enrichment (22, 23). Therefore, characterization of SM may be used as an important tool for assessing environmental health and monitoring changes in the ecological system (20, 24, 25).

Currently, meiofauna (meiobenthos: foraminifera, nematodes, and oligochaetes) are the recommended bioindicators of marine ecosystems and are also sensitive to contamination (26). Arguments advanced against these biomarkers underline difficulties in identification and the high frequency of sampling required. Furthermore, the effect of pollution on meiofauna, such as changes in abundance and diversity, changes in reproduction capability, and increased abnormalities (26, 27), take time to be observed and interpreted. These arguments further endorse the development of microbiota-based monitoring systems, which are relatively easy to sample and exhibit rapid responses to environmental changes and thus simplify monitoring efforts.

**FIG 1:**
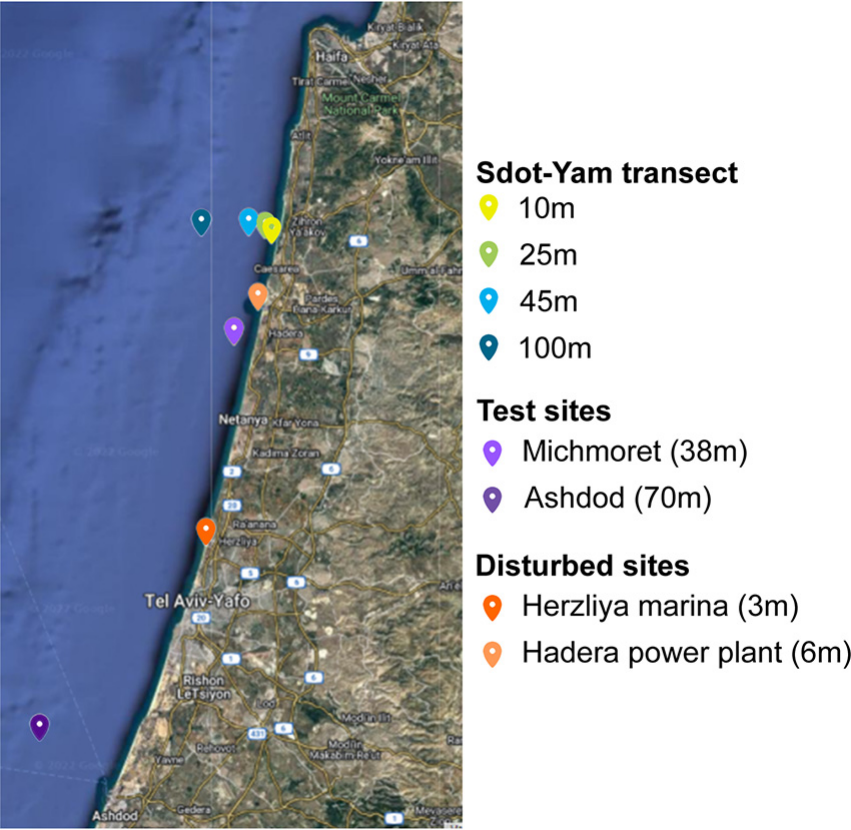
Map of the study site. Site coordinates are presented in Table S1.

The EMS continental shelf, specifically offshore from Israel, is characterized by a variation in geophysical and geochemical properties (e.g., grain size, organic matter content, trace metal concentration) along (north to south) and across (east to west) the shelf (28). Additionally, it is exposed to an assortment of human activities, including desalination plants, gas rigs, power stations (e.g., Hadera power plant), aquaculture, marinas (e.g., Herzliya marina), industrial and agricultural wastewater, recreation, and sports (Fig. 1). Currently, only a few studies describe SM at this region (29). Therefore, in this 3-year study, we sampled and analyzed sediment from relatively undisturbed and disturbed sites. The aims of the current study were to: (i) resolve environmental drivers of microbiota assembly along the Israeli continental shelf (10- to 100-m seafloor depth and 0- to 10-cm core-depth) with particular emphasis on cross-kingdom integration of information and (ii) provide a comprehensive characterization of the SM of this region and examine its applicability as a reference model for environmental changes and health.

## RESULTS

Marine sediment was sampled along one seafloor-depth transect (10, 25, 45, and 100 m) at the Sdot-Yam (SY) site from 2017 to 2020 (once each winter and summer; Fig. 1; see Table S1 in the supplemental material). In order to describe the SM, we analyzed *Archaea*, *Bacteria*, and *Eukaryota* composition by sequencing of 16S and 18S rRNA gene amplicons (Table S2).

**FIG 2:**
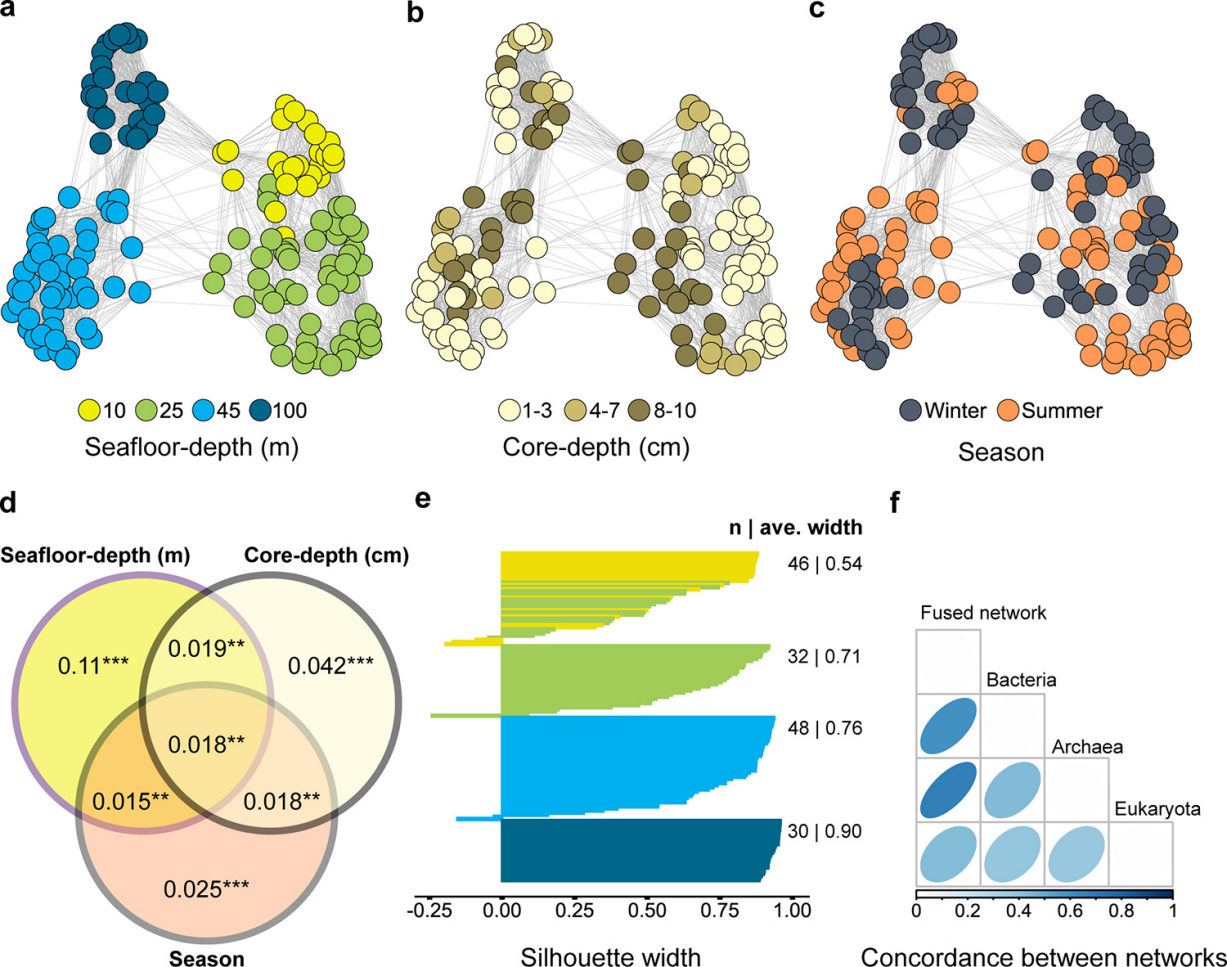
Continental shelf sediment microbiota exhibit robust microbiota composition controlled, across kingdoms, by seafloor depth. (a to c) Fusion network representing similarity in composition among the main samples from Sdot-Yam site (*n *= 156) based on information of *Archaea*, *Bacteria*, and *Eukaryota*. Samples are colored by seafloor depth (a), core depth (b), and season (c). (d) Permutational analysis of variance (PERMANOVA) testing significance and contribution to variance of explanatory variables and their interactions. **, *P* < 0.01; ***, *P* < 0.001. (e) Silhouette analysis support for the cluster number determined for the fused network. (f) Concordance matrix for fused network and data set of each kingdom.

First, we described the composition of SM along the SY seafloor-depth transect and how seafloor depth, core depth, and season influenced SM composition. This was done using a set of 156 main samples for which all three kingdoms were represented in our data set (Table S2). A fused similarity network of the three kingdoms demonstrated higher impact of seafloor depth on SM composition compared to core depth or season (Fig. 2a to c). We estimated the significance and contribution to variance of these three factors using permutational multivariate analysis of variance (PERMANOVA) performed with combined data from the three kingdoms. Together, the three parameters explained 24.7% of the variance, and the highest contribution was seafloor depth (11%), followed by core-depth (4.2%) and season (2.5%; Fig. 2d). A large part of the variance was explained by interactions among the factors tested (7%). This test was performed again using the data sets from each kingdom separately and revealed a similar trend (Table S4).

Based on the fused network, four clusters were highlighted and largely represented seafloor depth-based clusters. The four clusters averaged a silhouette score of 0.7, indicating strong structure (Fig. 2e). Concordance analysis, given the fused network and four clusters (Fig. 2f), indicated that the contribution of *Archaea* to the fused matrix was greatest (confidence interval [CI], 0.68), followed by *Bacteria* (CI, 0.6), and *Eukaryota* (CI, 0.42). These values pointed to a high contribution of data from each of the kingdoms to the final network and clustering. Indeed, similar network analysis for each of the three data sets produces much lower signal for all of the kingdoms, particularly for *Eukaryota* (Fig. S2). Thus, fusing data from all kingdoms benefitted our ability to detect and quantify the environmental effects on SM composition.

**FIG 3:**
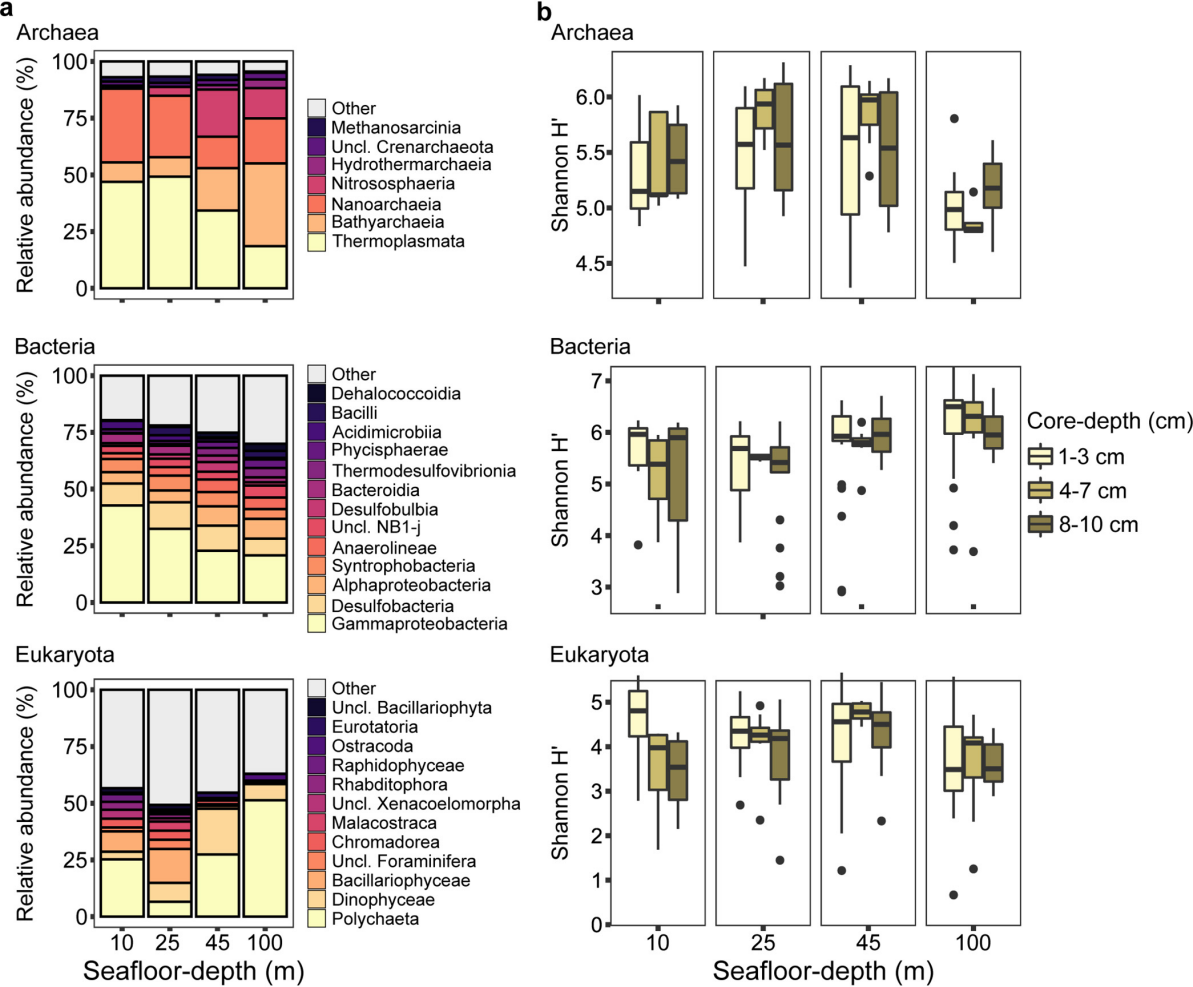
Composition and structure of sediment microbiota in the main samples. (a) Class-level composition. Means of relative abundances for classes for each seafloor depth are presented. Classes for which mean relative abundance was below 3% for any of the seafloor depths were summed and denoted as “other”. (b) Boxplot presenting the distribution of Shannon H′ index of diversity within each core-depth interval at each seafloor-depth site.

We then compared the composition and structure of SM along a seafloor-depth gradient. Within *Archaea*, four classes within three phyla (*Thermoplasmata*, *Crenarchaeota*, and *Nanoarchaeota*) accounted for 87% to 89% of the community in samples from all seafloor depths. However, the level of dominance of these classes varied greatly: at 10-m and 25-m depth, *Thermoplasmata* and *Nanoarchaeota* dominated; at 45-m and 100-m depth, two *Crenarchaeota* classes, *Bathyarchaeia* and *Nitrososphaeria* dominated (Fig. 3a).

The dominant group of *Bacteria* in the sediment was *Gammaproteobacteria*. The relative abundance (RA) of *Gammaproteobacteria* decreased with increased seafloor depth (43% at 10 m to 21% at 100 m). In contrast, the RA of two *Chloroflexi* classes (*Anaerolineae* and *Dehalococcoidia*) increased 1.6- to 19-fold between shallow and deep sites. Similarly, the RA of *Thermodesulfovibrionia* (*Nitrospirota*), bacilli (*Firmicutes*) *Phycisphaerae* (*Planctomycetota*), and *Alphaproteobacteria* were more abundant with depth, compared to shallow sites (Fig. 3b). Three classes of *Eukaryotes* were dominant in the sediment (Fig. 3c), each with highly variable RA at the different seafloor-depth sites. *Polychaeta* were the dominant *Eukaryota* at 100 m with 51% RA and least dominant at 25 m with only 6.6% RA. The *Dinophyceae* RA at 45 m was 2.4 to 6 times higher than in other sites. Lastly, *Bacillariophyceae* were dominant at 10 m and 25 m with 9% and 15% RA, respectively. Some specific classes showed high preference to a specific seafloor-depth site. For example, crustaceans of the class *Malacostraca* (*Arthropoda*) were dominant at 25 m (4%).

In order to assess differences in community structure, the Shannon index of diversity (Shannon H′) was calculated and compared in two factorial models. Model 1 compared seafloor depth and season; model 2 compared seafloor depth and core depth. Nonparametric factorial analysis based on aligned rank transformation (ART) tests for both models identified significant differences among seafloor-depth sites for *Archaea* (*F* = 11.16, *P = *0.000001), *Bacteria* (*F* = 8.04, *P = *0.00005), and *Eukaryota* (*F* = 8.61, *P = *0.00002) (Table S5). Only *Archaea* exhibited a significant effect across seasons (*F* = 25.88, *P = *0.000001) and for core depth (*F* = 4.26, *P = *0.016) (Table S5, Fig. 3b).

**FIG 4:**
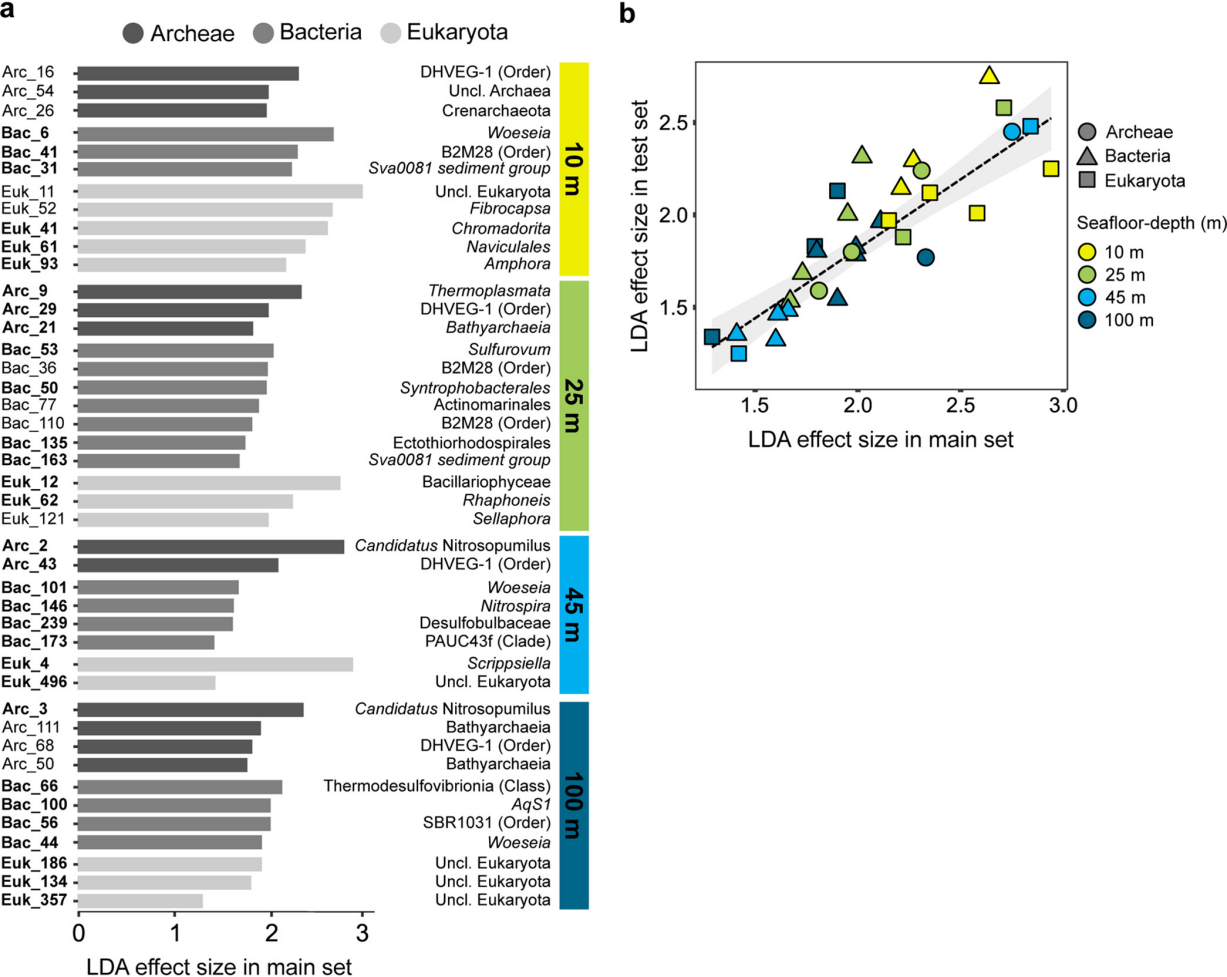
Microbial markers of seafloor depth in the main samples exhibit robustness. (a) Markers at the ASV level identified by the linear discriminant analysis effect size (LEfSe) methods (BH adjusted *P* < 0.05, LDA >1.25). Taxonomic assignment for each ASV is given up to the genus level. (b) Correspondence between LDA effect size in the main sample set and test sample set. LEfSe independently conducted for the test samples concurred about 32 of the 43 seafloor-depth markers (panel a) (FDR adjusted *P < *0.05, LDA >1.25). For the LDA effect size values of the 32 concurring markers: Pearson correlation coefficient = 0.87, *P < *0.0001.

We searched for microbial markers of seafloor depth using the main samples. A list of 43 amplicon sequence variant (ASV) markers was assembled based on linear discriminant analysis effect size (LEfSe) and fusion network analysis (Fig. 4a, Table S6). Some markers which shared taxonomic relatedness were assigned to different seafloor depths. For example, different archaeal markers belonging to the class *Thermoplasmata* (i.e., *Thermoplasmata* and DHVEG-1) were assigned to each of the seafloor-depth sites. In contrast, some taxonomic groups were represented by a single marker assigned to a specific seafloor depth. For example, *Nitrospira* (*Nitrospiria*) and *Sulfurovum* (*Campylobacteria*) were represented in 45-m and 100-m markers. Eukaryotic markers of seafloor depth represented the dominant classes, with distinct taxa assigned to each seafloor depth. In order to test the robustness of these microbial markers, we used the additional set of test samples collected for each kingdom (28 for *Archaea*, 39 for *Bacteria*, and 47 for *Eukaryota*) not included in the main set. For this test set of samples, we applied the LEfSe test. A total of 74% of the markers (32 of 43) verified the model’s estimation (Fig. 4a and b, Table S6 [marked in boldface in Fig. 4a]).

**FIG 5:**
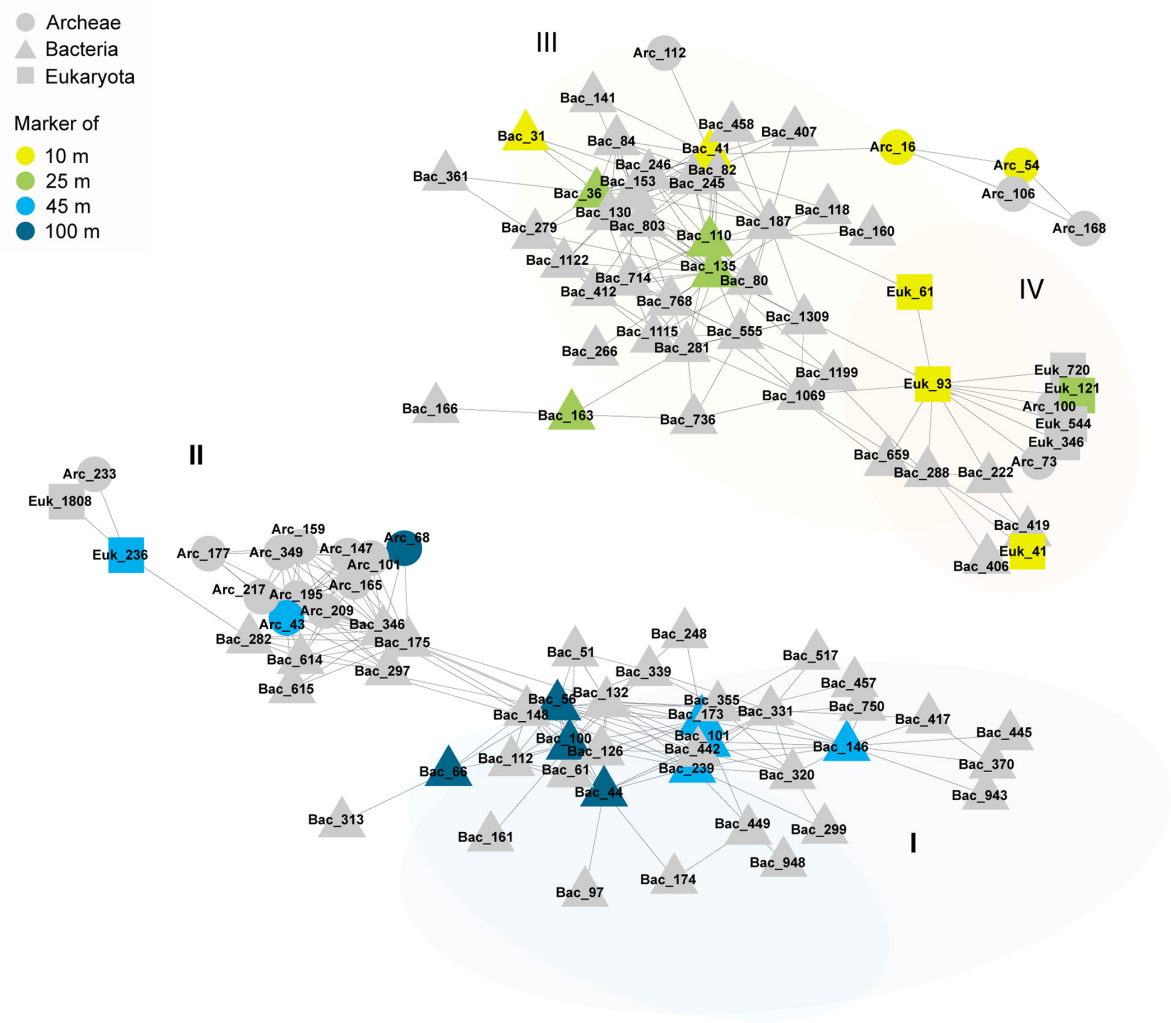
Cross-kingdom cooccurrence network based on association with seafloor-depth markers. Spearman correlations were calculated among ASVs across all data sets. Significant (*P < *0.05) and positive (Spearman rho ≥ 0.75) ASVs cooccurring with identified seafloor-depth markers were used to calculate the cooccurrence network.

As the list of seafloor depth-assigned markers represents the group most associated with this key environmental factor, this set of markers was used for detection of intra- and interkingdom associations. An association network of microbiota populations was constructed based on Spearman correlations among ASVs of all kingdoms, considering only significant correlations (*P < *0.05) and selecting only for positive correlations (Spearman rho > 0.75). The network included 119 ASVs; among those were 24 of the markers and 95 additional ASVs (Fig. 5). Two large clusters formed, one including the shallow seafloor-depth markers (10 to 25 m) and the second including the deeper seafloor-depth markers (45 to 100 m). Most of the network connections represented intrakingdom associations and were among bacterial ASVs (Fig. 5, clusters I and III), but interestingly, there were interkingdom connections (e.g., *Chromadorea* [Euk_41] and *Cytophagales* [Bac_406, cluster IV]).

**FIG 6:**
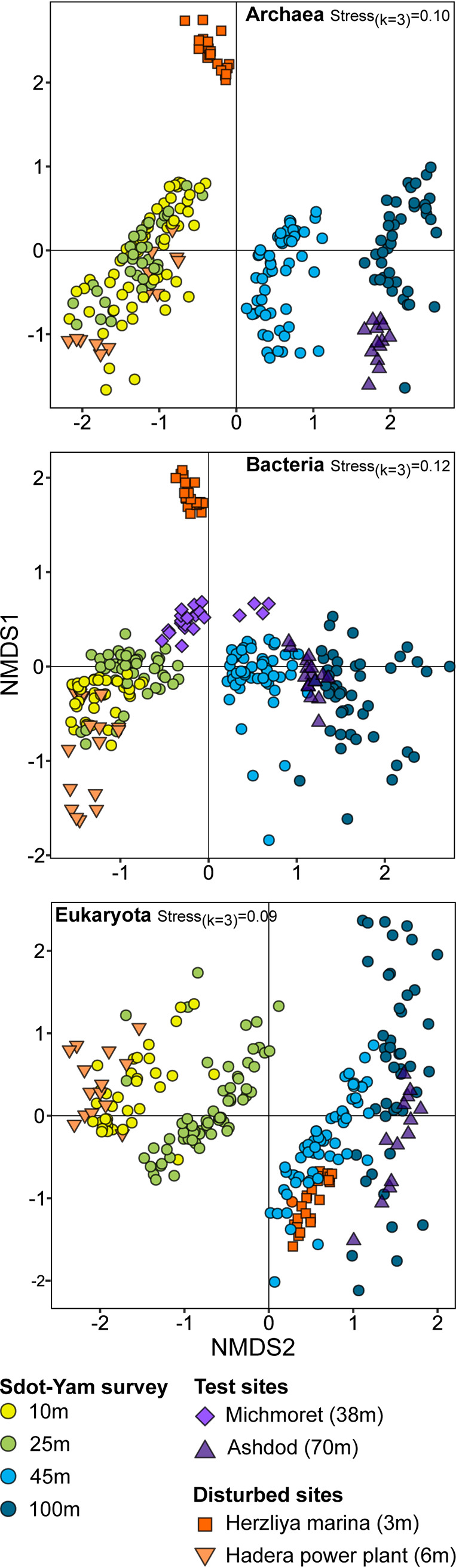
Similarity among sediment microbiota composition in model sites, test sites, and disturbed sites. Nonmetric multidimensional scaling analysis was calculated based on Bray-Curtis dissimilarities among samples.

The SY transect SM model across the three kingdoms showed stability of composition over 3 years of sampling (Fig. 2). Therefore, we examined the correspondence between microbiota at other regional sites, including other relatively undisturbed sites (the test sites, i.e., Ashdod 70 m and Michmoret 38 m) and highly anthropogenically impacted sites (disturbed sites, i.e., Herzliya marina [HM] and the Orot Rabin power plant in Hadera [HPP]) (Fig. 1). Nonmetric multidimensional scaling (NMDS) analysis was performed for *Archaea*, *Bacteria*, and *Eukaryota* separately, and respective ordinations were plotted (Fig. 6a, b, and c, respectively). Importantly, test sites selected for comparison were consistent with trends observed for SY across kingdoms, particularly regarding seafloor depth. Thus, the key role of seafloor depth in shaping SM is highly supported.

**FIG 7:**
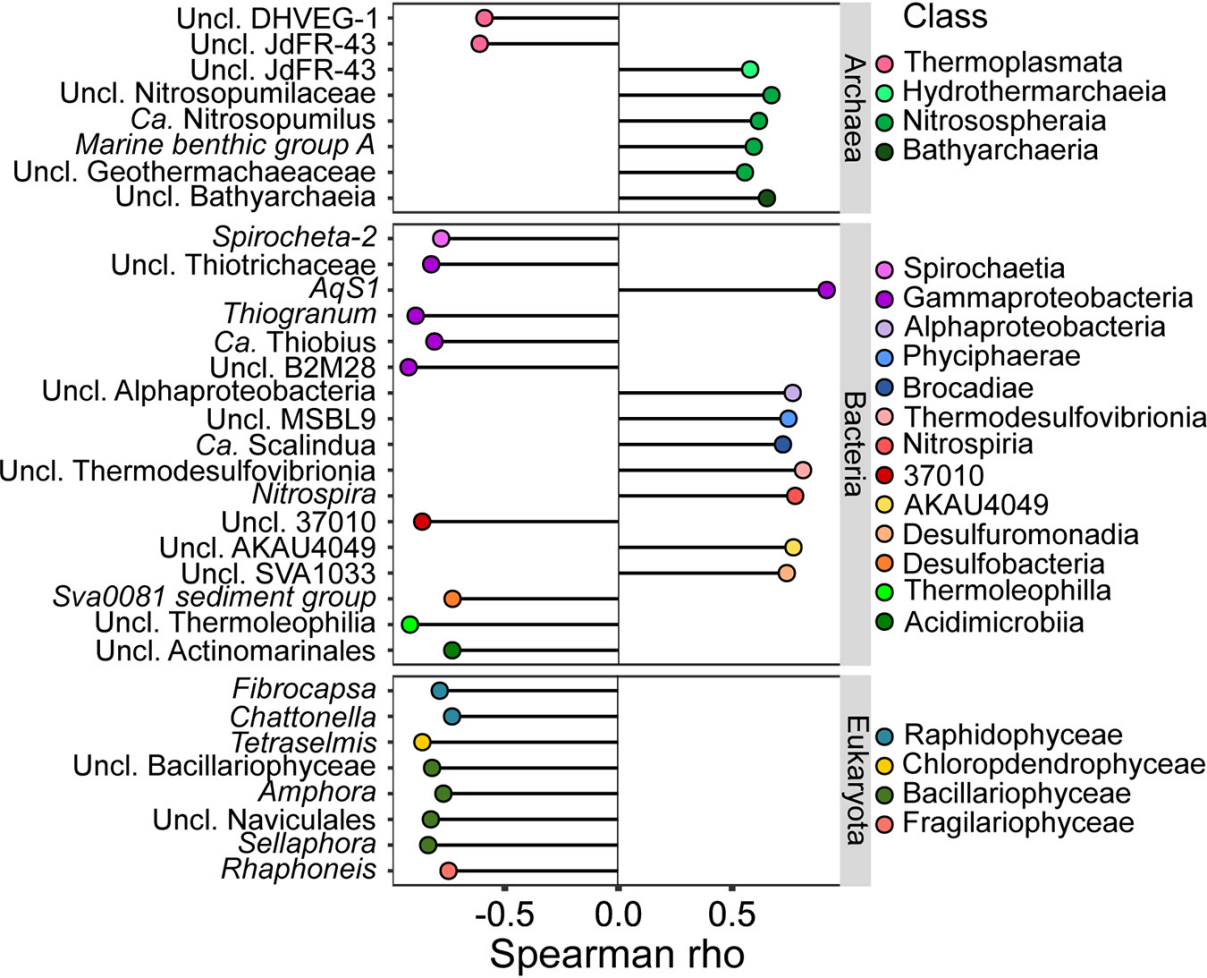
Spearman correlation between seafloor depth and microbiota members. Normalized count data, summed to the genus level, were used for calculation of Spearman correlation using the seafloor depth of 6 sites (model sites and test sites) as a predictor. Only significant correlations are presented (FDR adj. *P < *0.05). The threshold for Spearman rho was >0.5 for *Archaea* and >0.7 for *Bacteria* and *Eukaryota*.

To identify specific taxa which respond to seafloor-depth, we calculated Spearman correlations between seafloor depth and RA, after binning of ASVs up to the genus level. The main samples as well as test site samples were included (Fig. 7). Notably, the correlations were lower in *Archaea* than in *Bacteria* or *Eukaryota*. In order to select for the most prominent correlations, Spearman rho criteria were maintained to >0.5 for *Archaea* and >0.7 for *Bacteria* and *Eukaryota*. In *Archaea*, positive correlations were found between seafloor depth and four different groups within the class *Nitrososphaeria* and negative correlations with two different orders within *Thermoplasmata*. In bacteria, most of the correlated groups that belonged to *Gammaproteobacteria* were negatively correlated with depth. For *Eukaryota*, all the detected correlations were negative. Those included four different taxa of *Bacillariophyceae* (Fig. 3a).

**FIG 8:**
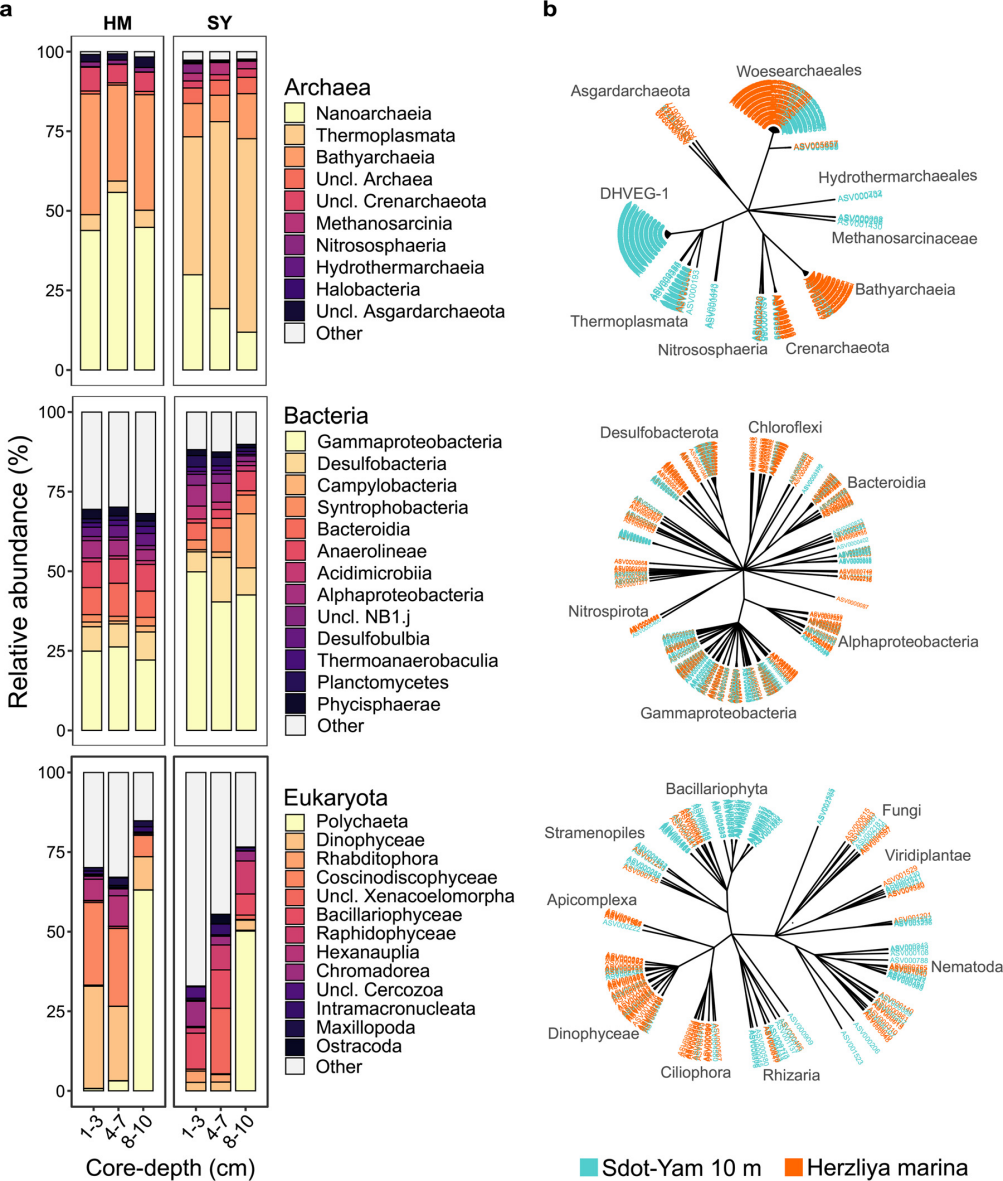
Disturbance impact on sediment microbiota composition. (a) Class-level composition at Herzliya marina (HM), compared to the model site at 10 m (SY). Means of relative abundances for classes for each site are presented. Classes for which mean relative abundance was below 3% depths were summed and denoted “Other.” (b) Cladogram showing taxonomic relatedness of markers at the ASV level identified by the linear discriminant analysis effect size (LEfSe) methods (FDR adjusted *P < *0.05, LDA effect size, >2.7; see Table S7).

As demonstrated by the NMDS ordination (Fig. 6), the SM of HM, a highly disturbed site, was markedly distinct in composition across all kingdoms. At HPP, the impact of disturbance was better resolved for *Bacteria* than the two other kingdoms. The HM site was further inspected for disturbance to microbiota composition. As this site was only sampled in winter, we compared the composition of those samples to the shallowest depth of the model sites (10 m), also sampled in winter. Figure 8a describes the composition of the microbial communities, including the three kingdoms and core depth. Figure 8b depicts taxonomic relatedness of ASV-level markers identified by the LEfSe procedure (adjusted [adj.] *P* < 0.05; linear discriminant analysis [LDA] score > 2.7; Table S7). Most dramatic were the changes in *Archaea* composition. For example, the RA of *Nanoarchaeota*, the dominant group, was 2.4 times higher at HM than SY. In contrast, class *Thermoplasmata* dominated SY with a 10-fold reduction in RA at HM. This class was represented by 93 markers, all but three assigned to SY. The *Thermoplasmata* order DHVEG-1 was missing completely at HM. Class *Bathyarchaeia* (*Crenarchaeota*) was enriched at HM (3.3-fold), with 35 of 40 markers for this group assigned to HM. *Dinophyceae* (*Alveolata*) exhibited a clear core-depth gradient at HM (32% at upper to 10% at deep core depth), where this group was dominant. A total of 35 *Dinophyceae* markers were identified, 28 of which were assigned to HM. Two other groups enriched with HM markers were the phyla *Ciliophora* (11 of 13 markers) and *Apicomplexa* (4 of 5 markers). Thus, clear markers of disturbance were readily identified.

## DISCUSSION

### Drivers of microbiota composition.

Biodiversity is the keystone of ecosystem functioning and environmental health. At that, microbiota comprise the foremost part of the biodiversity and provide the majority of ecosystem services (9). We obtained a high-spatial and -temporal resolution map of the microbiota along the EMS shelf transect. Integrating data across the three main kingdoms was key to resolving the main drivers of the SM assembly (Fig. 2, Fig. S2). Clustering of samples was highly supported in the integrated data set and much less so when considering each kingdom separately. Interestingly, the contribution of *Archaea* and *Bacteria* to the clustering of samples was higher than that of *Eukaryota*. This phenomenon might be explained by the high sensitivity ascribed to *Archaea* and *Bacteria* with respect to environmental conditions (30). Another explanation may be the difference in homogeneity of distribution of organisms within the sediment, with patchier distribution of *Eukaryotes*, particularly meiofauna or larger organisms, compared to prokaryotes (31, 32).

The continental shelf of Israel was characterized by several key properties, including particle size distribution, total organic carbon (TOC), water content and carbonate content (150 km north to south) for seafloor depths between 10 m and 100 m, attributing direct correspondence between seafloor depth and the measured parameters (28). Previous studies of marine and estuarine sedimentary systems show that the properties of sediment are key to determining the composition of microbiota (15, 32–35). Here, seafloor depth, representing these properties, explained 11% of the variance among samples (Fig. 2). Similarly, a survey of costal sediment of the Mediterranean Sea and French Atlantic Ocean estimated that grain size and TOC contribute 10% of the variance in microbiota composition (33). Second in importance was the core-depth variable, which explained 4.2% of microbiota variance among samples (Fig. 2). This significant effect can be attributed to dramatic shifts in the physicochemical conditions within the sediment (36, 37). Distinct physiologies and lifestyles may characterize microbiota even millimeters apart within the sediment core (37, 38). The factor of season was third in relation to its contribution to microbiota variation, amounting to only 2.5%. In sediment systems more exposed to the atmosphere (e.g., rivers, estuaries [39] coastal sands [40, 41], marine water column at shallow seafloor depth [42]) a large portion of the variation in microbiota composition was attributed to season.

### Variation in the composition of main groups of microorganisms is related to seafloor depth and core depth.

Major variation in the composition of all kingdoms was observed (Fig. 3a). *Gammaproteobacteria* and *Desulfobacteria* were the dominant bacterial classes in this study and were often found to be dominant in marine sediments (15, 36, 43, 44). One major observed effect on bacteria was the reduction in RA of *Gammaproteobacteria* from the shallow to deep sites. A compensative increase in RA was attributed to a large number of highly diverse populations, related to over 150 different classes within 61 phyla. In agreement, overall bacterial diversity significantly increased with seafloor depth (Fig. 3b, Table S5).

In *Archaea*, similarity in the RA of dominant classes was higher between 10 m and 25 m than between any other pair. This similarity may be explained by higher exposure to physical impacts such as waves or storms, and water mixing in shallow depths, or may reflect a dispersal barrier. The four main classes of *Archaea* are ubiquitous in marine sediment (15, 45). *Nitrosphaeria* perform ammonia oxidation, even at low concentrations, which characterizes the open ocean (46, 47) and the EMS, in particular (48). The RA of *Nitrososphaeria* was dramatically higher at the deep site, which is characterized by small grain size (Fig. 3a, Fig. S1). A study of estuarine sediments found negative relatedness between ammonia-oxidizing *Archaea* abundance, diversity, and grain size (49). The increase in *Nitrosphaeria* RA was accompanied by a proportional decrease in RA of the classes *Thermoplasmata* and *Nanoarchaeota*. The latter group is largely composed of organisms of reduced genome size that, based on current evidence, are considered to be obligate symbionts of other *Archaea* (45). Recent metagenomic surveys identified several *Nanoarchaeota* hosts from the class *Thermoprotei* of *Crenarchaeota* (48–52). However, *Nanoarchaeota* were first observed attached to *Archaea* of the order *Thermoplasmatales* (*Thermoplasmata*) in biofilm (53). It remains to be determined whether the *Nanoarchaeota* found here maintain a symbiotic lifestyle and, if so, what are their hosts.

*Polychaeta* was the dominant group of eukaryotes in the sediment (Fig. 3c) as in other marine and estuary sediments (54). The RA of *Polychaeta* was highly variable among sites, ranging from 6% (25 m) and 51% (100 m) with no direct correspondence to seafloor depth. This group plays a major role in the function of benthic communities, responsible for bioturbation of the sediment and burial of organic matter by their burrowing and feeding activity (55, 56). Therefore, their activity radically alters niche properties and, as a result, engineers microbiota assembly and structure (37, 57). Like the *Polychaeta*, patchy distribution characterized other dominant *Eukaryotes*. Those include *Dinophyceae* (*Alveolata*), *Malacostraca*, *Ostracoda* (*Arthropoda*), and *Eurotatoria* (*Rotifera*). These groups include many pelagic species that accumulate in the sediment following their death and sinking (58, 59). Thus, their patchy pattern may be related to abiotic and biotic processes in the water column.

### Model system for monitoring.

Biological indicators are considered preferable to hydrological or chemical surveys, as biological entities transmit an integrated and cumulative measure of ecosystem variations and/or disturbances, rather than a direct snapshot. Many marine studies have focused on the water column and have demonstrated the ability of environmental genomic surveys to detect environmental shifts and disturbances, even in real-time (60–62). However, biotic and abiotic factors vary temporally (e.g., day versus night) and along the water column (63, 64), and episodic events (e.g., contaminant spill) may dissipate rapidly in the water body, leaving no signature (65). Therefore, frequent water sampling is required to identify the disturbance or standardize sampling to represent specific aspects. In comparison, the sediment is more stable over time, is accumulative in its nature (66), and can register past events, even episodic ones (67). Considering overall effort, sediment monitoring may better serve local -to regional-scale regulatory needs.

Two approaches were examined to demonstrate the applicability of the SM model: (i) the specific markers and (ii) the whole community as a marker.

### Specific-marker approach.

Many studies attempt to identify specific microbial markers of environmental status based on limited (even a single snapshot) sampling events. However, in order to obtain robust markers, sampling must be repeated to reach stability and assess reproducibility (65, 68). The robustness of our SM model was demonstrated by high reproducibility of specific markers for seafloor depth between the main samples and a set of test samples collected over the study period (Fig. 4).

The marker approach can be further expanded to incorporate not only a set of markers, but also cooccurrence patterns. The cooccurrence network that included seafloor-depth microbial markers demonstrated both intra- and interkingdom connections (Fig. 5). For example, a strong positive correlation between a nematode marker (Euk_41) and a *Cytophagales* population (Bac_406) was demonstrated (Fig. 5, cluster IV). These two groups were found to be codominant in a previous study of marine costal sediments (13). Cooccurrence of species may reflect shared ecological filtration (i.e., selection) or codependency (e.g., symbiosis). In addition, stochastic processes, such as dispersal limitation, are also manifested by high cooccurrence, as was demonstrated for archaeal communities in coastal sediment (69) and is indicated in the network here. For this reason, examination of cooccurrence networks is appealing, particularly in cross-kingdom data sets. Previous studies have used microbial cooccurrence networks to resolve between different biotic or abiotic conditions (70–73). Hence, analysis of cooccurrence patterns may be useful in detecting changes and perturbations. An additional set of markers was identified by direct analysis of correlation between seafloor depth and RA of different taxa (Fig. 7). This approach may be applicable for gradients in environmental conditions. In this setup, the relevant underlining gradients are sediment grain size and TOC. In order to improve the sensitivity and reproducibility of markers, continued sampling and model refinement are required. Furthermore, specificity of the markers and reliability for biomonitoring may be improved by application of more advanced computational methods, such as machine learning, that were shown to outperform more traditional methodologies, including indicator value and random forest analysis (74).

### Whole-community approach.

While providing a robust mean for classification of samples, the biomarker or specific marker approach is limited in the sense that it absolutely relies on prior knowledge and assumes a steady state. Natural habitats change over time, with or without perturbations, affecting the validity of any marker. Furthermore, specific markers may not be affected by one or another type of perturbation, disabling the detection of an incident or a process. A whole-community approach may therefore have an advantage, as it enables identification of changes over time (30) and relies on measurement of similarities rather than *a priori* determined criteria. Whole-community examination of SM by NMDS analysis (Fig. 6) demonstrated the applicability of this approach. Samples collected at the test sites were ordinated in a manner expected based on their seafloor depth. Naturally, detection of disturbances is a key goal for monitoring efforts. Samples collected from HM, a highly disturbed site, were markedly divergent from model sites and across the three kingdoms (Fig. 6). Marinas are a common perturbation to the marine environment, typically physically confounded, reducing water circulation, intensely and chronically impacted by diverse anthropogenic activities. Among common pollutants in marinas are organic matter, particularly oil and oil products (75, 76). The intensity and diversity of pollutants was reflected in SM composition (Fig. 8, Table S7). *Ciliophora* and *Dinophyceae*, previously associated with organic pollution and oil pollution (77, 78), were higher at the marina than the model site. Similarly, *Gammaproteobacteria* and *Bacteroidetes* that increased in RA at the marina were previously identified as dominant bacteria in a chronically oil-contaminated lagoon (79). *Anaerolinea* (*Chloroflexi*) was denoted for its contribution to tetrabromobisphenol A dissipation in mangroves (80). *Asgardarchaeota*, identified as a marker of HM, was previously reported to be associated with petroleum hydrocarbons and polychlorinated biphenyls (81).

**Conclusions.** This study examined the suitability of marine SM as a bioindicator for environmental health in monitoring programs of the EMS. The main factor determining microbiota composition in EMS costal shelf sediment was the seafloor depth, which in this context, represents basic sediment characteristics: grain size and TOC. This enabled a robust and sensitive model that is applicable for sites outside the monitoring stations and that detects disturbance. Identification of robust markers was feasible. However, further study is required in order to validate them, understand their ecological role, and elucidate their mechanisms that determine niche preference.

## MATERIALS AND METHODS

### Study site.

Sediment was sampled twice a year (winter and summer) for 3 years (2017 to 2020) along a four-seafloor-depth transect (10, 25, 45, and 100 m) near Sdot-Yam (SY), an undisturbed site (Fig. 1, Table S1). These four sites are termed model sites in this article. To further expand and establish the model, additional sites were selected, Ashdod and Michmoret (70 m and 38 m seafloor depth, respectively), designated the test sites and were characterized by their low anthropogenic impact. Additionally, sediments were taken at Herzliya Marina (HM) and the Orot Rabin power plant in Hadera (HPP) (3 m and 6 m seafloor depth, respectively) since these sites experience high rates of anthropogenic impact and are designated disturbed sites (Fig. 1, Table S1). For model sites, the total organic carbon (percent TOC) and grain size were measured, as previously described (25, 82) (Fig. S1). For further description of the study sites, see File S1.

### Sampling procedure.

Two cores (6-cm diameter, 20 cm long) from each seafloor depth were hand-sampled during SCUBA dives from 10- to 45-m depths or sampled using a box corer from 100-m depth. The samples were chilled until transfer to the laboratory. There, each core was split to 1-cm slices, up to 10-cm core-depth, with sterile tools. The slices were kept in separate tubes at −20°C until further processing. Table S2 lists the samples used in this study.

### DNA extraction, PCR amplification, and amplicon sequencing.

Samples were processed directly following each sampling event. DNA was extracted using the DNeasy PowerSoil kit (Qiagen, Valencia, CA, USA) following the manufacturer’s instructions. Appropriate controls were included in all parts of sample preparations (no input reactions). For *Bacteria* and *Eukaryota*, primers were used for PCR amplification of small-subunit (SSU) rRNA gene fragments as described in The Earth Microbiome Project (83, 84). For *Archaea*, the primer pair used was published in Takahashi et al. (85). Please see File S1 for more details. The raw sequence data are available in the NCBI SRA database under BioProject accession PRJNA910589.

### Sequence data processing.

Sequence data were processed using the DADA2 pipeline (86). For each sequencing run, a separate analysis was conducted for quality trimming, error model estimation, sequence error correction, and amplicon sequence variant (ASVs) inference and quantification. The ASVs and count tables of all runs were merged, and suspected chimeras were removed. For each ASV, taxonomy was inferred by alignment to the Silva nonredundant small-subunit rRNA database (v138) using the DADA2 command assignTaxonomy, setting the minimum bootstrap value to 80%. For *Eukaryota* ASV sequences, taxonomic inference was achieved by the last common ancestor method (LCA) in MEGAN (MEGAN6, community addition) using as input results of a BLASTn search against the NCBI nucleotide database with the 50 best hits (LCA parameters: minimum score, 100, E-value, <10^−9^; a coverage, >80%). Table S3a to c present the raw count tables and ASV inferred taxonomy.

### Similarity network fusion for main samples.

Out of a total of 218 samples taken from the Sdot-Yam site, we obtained full high-quality results, including *Archaea*, *Bacteria*, and *Eukaryota* representation for 156 samples (Table S2). This set of samples, here termed main samples, served for the analysis of microbiota composition and structure. Samples from the Sdot-Yam site for which only partial data were obtained (i.e., not all three kingdoms) are here termed test samples. In order to take advantage of information resulting from the three data sets per sample (i.e., *Archaea*, *Bacteria*, and *Eukaryota*), we chose the similarity network fusion (SNF) approach as implemented in the SNF tool (87) For SNF analysis, each of the data sets was filtered to retain only ASVs detected in at least 10 of the 156 samples. For each data set, counts were log-ratio-transformed, and a distance matrix was calculated. The R package SNFtools (v2.3.1) was used for the calculation of affinity matrices from the distance matrices (k = 15, sigma = 0.5) and the fusion network. The number of clusters supported by the fused network was calculated by the “rotation cost best” method, and a subsequent concordance matrix was calculated. Silhouette analysis was also performed to support the chosen number of clusters (R package cluster [v2.1.3]). For comparison, networks were calculated from each of the data sets independently, using the same methodology. Additionally, using the fused network (Fig. 2), the ASVs from each kingdom were ranked by their normalized mutual information (NMI). The top 10 ASVs for each kingdom at seafloor depth were recorded as markers of the network clusters.

### Additional similarity estimates.

To examine the contribution of the environmental factors (seafloor depth, core depth, and season) to variation in microbiota composition in the main samples, permutational analysis of variance (PERMANOVA) was performed (command adonis in the R package vegan [v2.5.7]) based on Euclidean distances on log-ratio-transformed counts. Additionally, for model and auxiliary samples in each data set, nonmetric multidimensional scaling (NMDS) analysis was performed. NMDS was based on Bray-Curtis dissimilarities, calculated after cumulative sum square normalization of raw count data.

### Community structure.

For each of the three data sets, raw counts were subsampled to the minimum library size, and then a Shannon index was calculated (diversity function in the R package vegan). In order to test differences in Shannon index as a function of the environmental variables, aligned rank-transformed (ART) analysis of variance (ANOVA) was performed (88). As some of the season or the core-depth levels were missing in the model main sample data set, two models were tested: (i) Shannon ~ seafloor-depth × core-depth and (ii) Shannon ~ seafloor-depth × season. Differences were considered significant for a *P* value of <0.05.

### Differential abundance estimation and marker identification.

Linear discriminant analysis effect size (LEfSe) analysis was chosen to calculate differential abundance and identify putative markers. This method is effective in determining which features, in this case ASVs, are most likely to explain observed differences among factor levels (89). LEfSe was performed using the online Galaxy module (http://huttenhower.sph.harvard.edu/galaxy) for three sets of data: (i) main samples, (ii) test samples (in total, 62 samples; 28 *Archaea*, 39 *Bacteria*, and 47 *Eukaryota*), and (iii) disturbance set-HM versus main samples from 10-m seafloor depth. In all cases, Cumulative Sum Scaling (CSS) normalized count data were used as input. LEfSe was applied to each of the three sets separately. The factors seafloor depth (as main class) and core depth (as subclass) were used for detection of putative markers for seafloor depth. Test parameters were set to default, but the LDA effect size threshold was set to 1.25 for the main and test sets of samples and to 2.7 for the disturbance set of samples. For LEfSe results of the disturbance set, cladograms linking ASV markers based on taxonomic relatedness were drawn using the R package igraph (v1.2.0).

### Cooccurrence network.

For the set of identified markers for seafloor depth (above), cooccurrence with additional ASVs from all three kingdoms was examined. For this purpose, Spearman correlations were calculated between this set of ASVs and all other ASVs. Significant (false-discovery rate [FDR]-adjusted *P < *0.05) and strong positive correlations (Spearman rho ≥ 0.75) were selected and combined to provide a list of associated ASVs. Then, Spearman correlations among all ASVs in the combined list were calculated. Only significant correlations (*P < *0.05, Spearman rho ≥ 0.75) were maintained. An affinity matrix was then calculated based on this data set and used to create an association network using the R package igraph.

### Spearman correlations with seafloor depth.

Spearman correlations were calculated using count data from all samples excluding the disturbance set, summed to genera, and log-transformed. Spearman correlations were calculated and tested using the command rcorr in the R package Hmisc (v4.6.0). Correlations were considered significant with the Benjamini-Hochberg FDR adjusted *P* value was <0.05.

### Data availability.

The data sets generated and analyzed during the current study are available in the NCBI SRA repository, under accession number PRJNA910589.
