# Supplementary material for: Sediment Microbiota as a Proxy of Environmental Health: Discovering Inter- and Intrakingdom Dynamics along the Eastern Mediterranean Continental Shelf
Source: Microbiol Spectr. 2023 Jan 16;11(1):e02242-22. doi: 10.1128/spectrum.02242-22 (PMC9927165; doi:10.1128/spectrum.02242-22)
Supplement: Supplemental file 5 — Supplemental material. Download spectrum.02242-22-s0005.pdf, PDF file, 1.1 MB [file spectrum.02242-22-s0005.pdf]

Supplementary file 1: Supplementary methods, figures and tables.

Study site

Along the Israeli coastline and continental shelf there is high density of anthropogenic activity. Accordingly, much of the anthropogenic influence is complex due to multiple sources of disturbance. For the selected undisturbed site, there is minimal anthropogenic influence, mainly sparse movement of small boats. The HPP site, in comparison, is exposed to disturbances from four main sources: 1) Warm water effluents from the Hadera power plant; 2) Effluents from a desalination plant, causing increased water salinity; 3) Effluents from Hadera stream estuary. Rate of effluents is not constant and they include wastewater (treated and raw sewage), agricultural waste and surface runoff. 4) intense sporting activity, including boating, diving, swimming and more. The sampling site itself adjacent to the center of complex of disturbances, therefore not directly exposed. The Herzliya Marina (HM), like other marina, is intensely exposed to heavy boating and fishing and other recreational activity. The inputs include oils, organic wastes, diverse plastic compounds and in addition, intense light and noise disturbances. Samples were taken within the HM site itself.

DNA extraction and PCR amplification and amplicon sequencing

Samples were processed directly following each sampling event. DNA was extracted from each slice using the DNeasy powerSoil Kit (Qiagen, Valencia, CA, United States) follwoing manufacturer instructions. Appropriate controls were included in all parts of sample preparations (no input reactions). For Bacteria and Eukartyota, universal primers were used for PCR amplification of SSU rRNA gene fragments, as described in The Earth Microbiome Project (EMP)[1,2]. For Archaea, primer pair was as in Takahashi *et al*. (2014)[3]. The primers contained 5' common sequence tags (known as common sequence 1 and 2, CS1 and CS2)[4]. Amplicons were generated using a two-stage PCR amplification protocol[5]. Cycling conditions for the first stage PCR were 95°C for 5 minutes,

followed by 28 cycles of 95°C for 30 sec, 55°C for 45 sec and 72°C for 60 sec. Subsequent steps were carried out at Genome Research Core (GRC) at the University of Illinois at Chicago (UIC), USA. Samples were barcoded each with a unique 10-base barcode in a second PCR amplification (Access Array Barcode Library for Illumina, Fluidigm, South San Francisco, CA, USA; Item# 100-4876). Libraries were then pooled and sequenced with a 15% phiX spike-in on Illumina Miniseq flow cell (2x153 paired-end reads) for Bacteria and Eukaryota and Illumina MiSeq flow cell (2x250 paired-end reads) for Archaea. Raw sequence data was deposited in the NCBI SRA database under bioproject accession PRJNA847589.

References

1. Caporaso JG, Lauber CL, Walters WA, Berg-Lyons D, Lozupone CA, Turnbaugh PJ, *et al*. Global patterns of 16S rRNA diversity at a depth of millions of sequences per sample. PNAS, 2011;108:4516–4522.
2. Stoeck T, Bass D, Nebel M, Christen R, Jones MDM, *et al*. Multiple marker parallel tag environmental DNA sequencing reveals a highly complex eukaryotic community in marine anoxic water. Mol. Ecol. 2010;19:21–31.
3. Moonsamy PV, Williams T, Bonella P, Holcomb CL, Höglund BN, Hillman G, *et al*. High throughput HLA genotyping using 454 sequencing and the Fluidigm Access Array™ system for simplified amplicon library preparation. Tissue antigens, 2013;81(3):141-149.
4. Naqib A, Poggi S, Wang W, Hyde M, Kunstman K, Green SJ. Making and sequencing heavily multiplexed, high-throughput 16S ribosomal RNA gene amplicon libraries using a flexible, two-stage PCR protocol. In Gene expression analysis. Humana Press, New York, NY. 2018 pp. 149-169.
5. Takahashi S, Tomita J, Nishioka K, Hisada T, Nishijima M. Development of a prokaryotic universal primer for simultaneous analysis of Bacteria and Archaea using next-generation sequencing. PloS one, 2014;9(8):e105592.

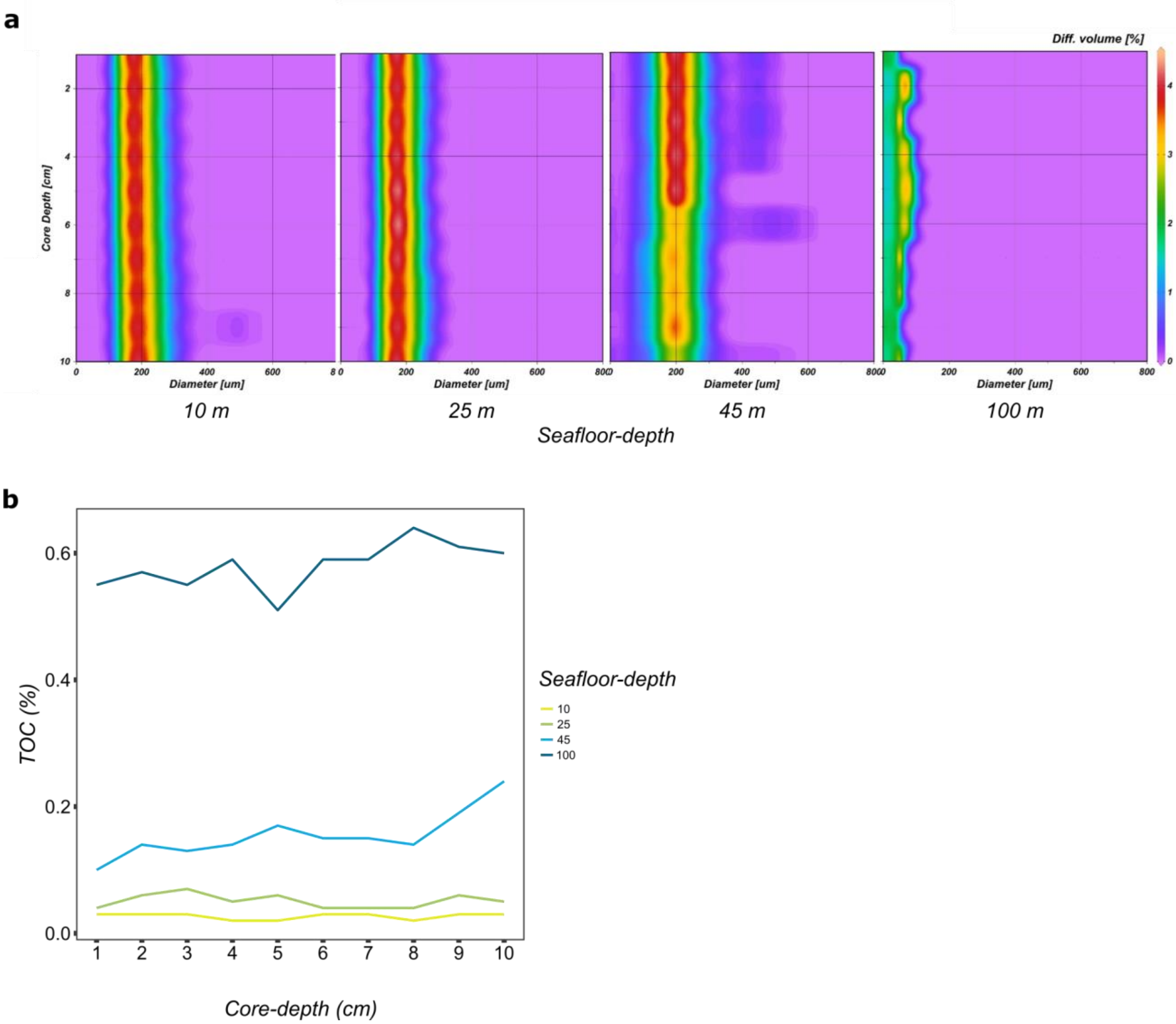

Fig. S1 Sediment properties along the model sites (10, 25, 45 and 100 m) and core-depths (1-10 cm). Relative volumetric grain size distribution in the sediments (**a**) and Total Organic Carbone (TOC) (**b**).

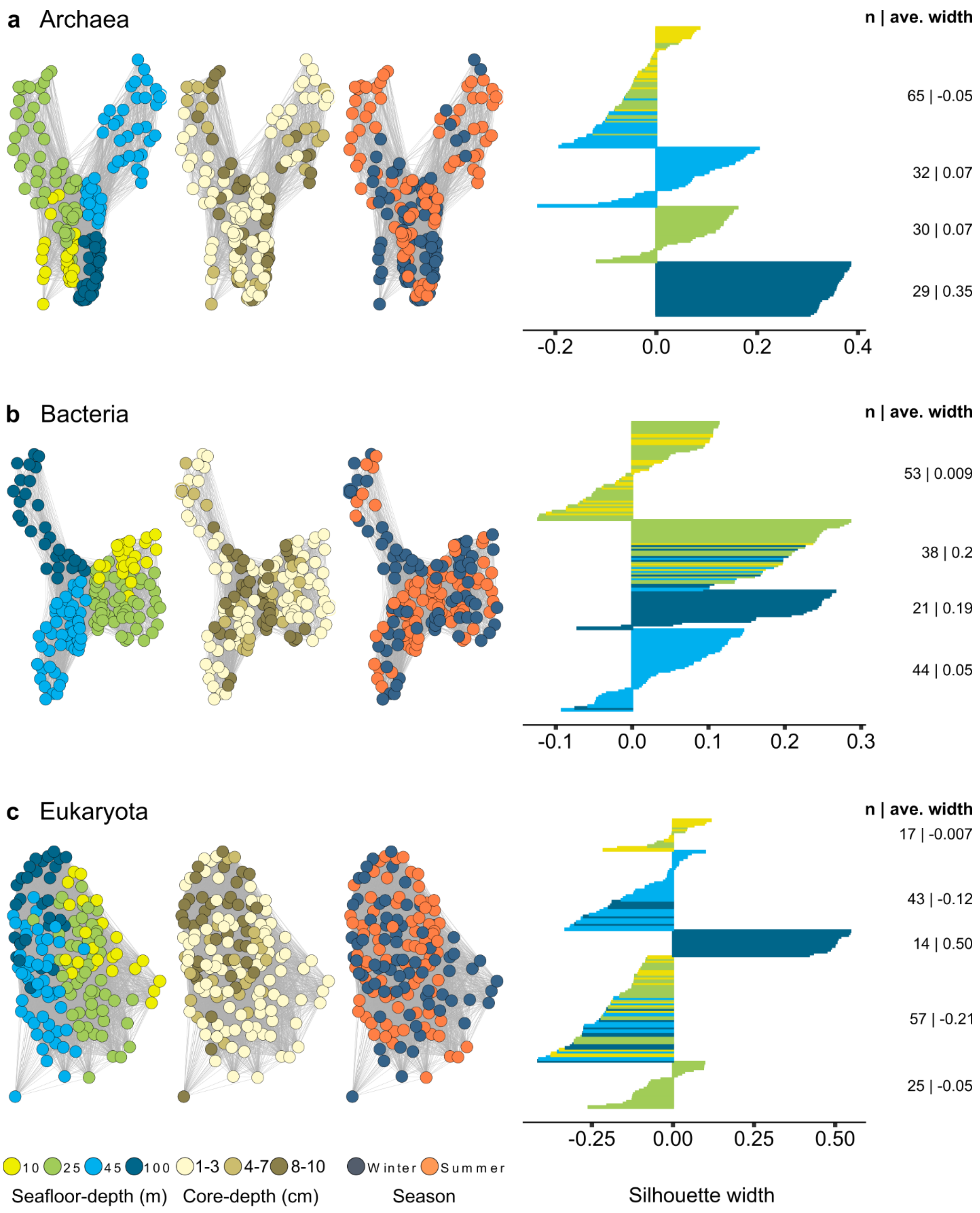

Fig. S2 Network analysis for main samples and silhouette analysis of sample clustering using information from Archaea (**a**), Bacteria (**b**) and Eukaryota (**c**) individually.

Table S3 Taxonomy and raw read counts for Archaea (a) Bacteria (b) and Eukaryote (C) samples.

The tables S3 a-c were upload on FigShare site. 10.6084/m9.figshare.20191130

Table S4: Impact of seafloor-depth, core depth and season on sediment microbiota composition. PERMANOVA was used to assess the significance and contribution of each factor and their interactions in Archaea, Bacteria and Eukaryota.

	**PERMANOVA *R^2* value**		
Factor	Archaea	Bacteria	Eukaryota
Season	0.021**	0.027**	0.024**
**Seafloor-depth (SD)**	**0.1****	**0.168****	**0.067****
Core depth (CD)	0.037**	0.053**	0.032**
Season x SD	0.02**	0.019**	0.015**
Season x CD	0.005	0.013	0.01**
SD x CD	0.015**	0.026**	0.016**
Season x SD x CD	0.006	0.009	0.009**

Table S5: Aligned rank transformed ANOVA test for Shannon H' index of diversity for sediment microbiota at SY site.

		**ART *F* value**		
		Archaea	Bacteria	Eukaryota
**Model 1**	Seafloor-depth (SD)	11.16***	8.04***	8.61***
	Season (S)	25.88***	0.69	0.04
	SD x S	4.29**	2.11	1.12
**Model 2**	Seafloor-depth (SD)	8.86***	8.53***	9.93***
	Core-depth (CD)	4.26*	1.41	1.64
	SD x CD	0.82	0.74	2.03.
